# Enhancing automotive cooling systems: composite fins and nanoparticles analysis in radiators

**DOI:** 10.1038/s41598-024-52141-0

**Published:** 2024-02-13

**Authors:** R. Ramesh Kumar, K. Karthik, P. V. Elumalai, R. Elumalai, Davannendran Chandran, E. Prakash, Nasim Hassin

**Affiliations:** 1 Vel Tech Rangarajan Dr.Sagunthala R&D Institute of Science and Technology, 400 Feet Outer Ring Road Avadi, Chennai, Tamil Nadu India; 2 Department of Mechanical Engineering, Aditya Engineering College, Surampalem, Andhra Pradesh India; 3 Department of Mechanical Engineering, Vellore Institute of Technology, Chennai, India; 4 Department of Mechanical Engineering, Universiti Teknologi PETRONAS, Seri Iskandar, Malaysia; 5 Department of Mechatronics Engineering, Rajalakshmi Engineering College, Chennai, India; 6 Department of Mechanical Engineering, Mettu University, Metu, Ethiopia

**Keywords:** Environmental impact, Mechanical engineering

## Abstract

Composites are driving positive developments in the automobile sector. In this study investigated the use of composite fins in radiators using computational fluid dynamics (CFD) to analyze the fluid-flow phenomenon of nanoparticles and hydrogen gas. Our world is rapidly transforming, and new technologies are leading to positive revolutions in today’s society. In this study successfully analyzed the entire thermal simulation processes of the radiator, as well as the composite fin arrangements with stress efficiency rates. The study examined the velocity path, pressure variations, and temperature distribution in the radiator setup. As found that nanoparticles and composite fins provide superior thermal heat rates and results. The combination of an aluminum radiator and composite fins in future models will support the control of cooling systems in automotive applications. The final investigation statement showed a 12% improvement with nanoparticles, where the velocity was 1.61 m/s and the radiator system’s pressure volume was 2.44 MPa. In the fin condition, the stress rate was 3.60 N/mm^2^.

## Introduction

Composite materials' usage is beneficial in the modern world. These materials are always lightweight and have more strength compared to other materials. This investigation study focuses on reducing the temperature effects from the radiator pipelines. The complicated investigation study is ongoing worldwide. Many countries are working hard to improve the utilization of composite materials effectively. This research focuses on fins-based composite materials and the results of using Nano-particles fluid. Nano fluids help to reduce the heat reductions and enhanced the materials life for the heat conduction. Mostly the heat radiators are getting failure its materials properties. Ultimate aim is to maintain the materials life properties and reduces corrosion rates. Increasing the life of radiators and not affected by the temperatures. So many of the researchers have done the works based on the different techniques and different strategies. Here below collected some literature review and ideas, problem conditions, different results. From the literature we can get good ideas for our problem conditions.

In their study, Karki et al.^1^ conducted a fluid investigation on all flow conditions and structures, with a focus on Nano fluid properties. They found that the molecular shapes and behaviors depended on mesh areas and coefficient of friction, and that the addition of Nano fluids decreased adjacent wall temperature levels. In the present study, composite fins and Nano fluid flows were used, resulting in increased heat transfer rates from the carburetor pipes. This is in comparison to previous studies that utilized only Nano fluids.

The fluid intermediate is affected by nano conditions and pressure levels. The successful cooling rate is achieved within 9 s from the inlet to the outlet. The magnification and pressure gradient are influenced by the boundary conditions. The structures and shapes of the curved pipes are prone to deformation, which affects the velocity levels. The velocity levels decrease from the inlet pipe to the outlet pipe, with higher values at the inlet and lower values at the outlet.

Ramadhan et al.^2^ investigated how inner flow pressure affects the shapes of radiator pipes and structures. They analyzed the complex surface shapes using CFD investigations and found that using composite fins with Nano fluid produced favorable results. The study took into account the temperature distributions and structural characteristics of the materials used.

In their study, Sultan et al.^3^ analyzed the impact of Nano fluids on the velocity and thermal interfaces in the curved areas of radiator pipes. They found that the velocity magnitude was primarily affected by Nano fluids, resulting in quick thermal interfaces. The cooling systems influenced the velocity magnitude, with the fins' contact areas cooling the ambient temperature. The study also revealed that stress occurred in the curved areas, with good linear contact and temperature inside the pipe flow. The Nano fluid interface changed from high to low temperatures. The thermal formulations and boundary conditions were kept constant throughout the experiments.

Zanzote^4^ investigated the contact of fin elements and their effect on energy consumption, comparing them with the radiator pipe. The temperature of the heated walls was found to be high, whereas that of the fins was very low during the CFD investigations. The thermal range inside the pipe was higher compared to the outer surfaces. The thermal stresses and cooling rate conditions were found to be higher compared to curved pipes. The thickness of the fins was found to be crucial in transmitting energy from the inlet walls. From the literature, it is clear that there are very limited studies on CFD studies related to composite fins employed in radiators. In this investigation, composite fins used in radiators were analyzed. The entire thermal modeling operations of the radiator, as well as the composite fin arrangements with stress efficiency rates, were studied in detail. This study explored the radiator's velocity path, pressure changes, and temperature distribution.

Hema Kumar and Senthilkumar^5^ compared the structural arrangements of nanoparticles, which were affected by the temperature changes and controls. They used CFD investigations to analyze the molecular structures and shapes. As the iterations increased in the CFD conditions, it eliminated the molecular behaviors and shapes. The authors chose the content of the molecular ranges and thermal effects to achieve a good stress rate from the surfaces. The angles of the curved structures were predetermined from the heat transfer controls.

Nabil et al.^6^ analyzed the heat transfer rates and structural shapes of radiators using CFD investigations. The study also examined the strain and stress values of heated pipes. Comparing air and water inlet radiator processes, the study found that the latter resulted in lower internal stress and strain rates. The complex heat structures and cooling systems were crucial in determining thermal behaviors. One common mistake in the CFD process is the occurrence of overheating temperatures. The cooling systems of natural composite structures were adopted from good natural surfaces. The composite results demonstrated better cooling systems and contact behaviors from the fins and radiators.

Vaidhilingam^7^ conducted a CFD simulation to compare the heat transfer rates of water and silver nitrate Nano fluids. The study focused on the effect of Nano fluid content on water flow. The initial velocity within the radiator was high, causing the temperature to vary from the inlet to outlet valves. Instead of using fins in the cooling system, fins were placed in contact between the valve fractions. Kumar et al.^8^ analyzed the energy conditions and found that the heat transfer rate was higher for Nano fluids compared to water.

Ponangi et al.^9^ investigated composite fins and their use in cooling systems for carburetor pipes. It was found that composite materials had higher temperature absorption levels than aluminum alloys, and that nitrogen oxides could be used as a cooling system for composite structures. The use of zinc particles on the surface areas of heated pipes to regulate temperature is also an innovative approach. The investigation by Oliveira et al.^10^ that the use of meshed elements and vector paths is a more complex approach to study heat transfer in composite fins." It should be "The investigation by Oliveira et al.^10^ showed that the use of meshed elements and vector paths is a more complex approach to study heat transfer in composite fins.

Kirubagharan et al.^11^ and Hatami et al.^12^ conducted studies on the geometric structure of radiators and the effects of temperature and cooling systems. The majority of the structure interface was found to occur at high-temperature gradients, with fewer occurrences at contour interfaces. In both studies, cooling arrangements were implemented for the fins and water particles. Oval and circular tubes were utilized for the investigation, and it was found that the temperature effect was greater in the oval tube as compared to the circular tube. Copper-brass fins were used in the radiators for both studies.

Akash et al.^13^ analyzed the thermal heat energy absorption characteristics of composite fins. The study used banyan and epoxy resin to form the composite fin structures. The adequate strength of banyan achieved high epoxy controls in natural cooling systems. The composite materials absorbed heat more quickly compared to normal materials. The process and behavior of natural fibers were found to be very efficient.

Tomar and Sharma^14^ studied the effects of pressure on radiator pipes and found that the intensity of Nano fluids was higher than that of normal fluids, which was derived from the microstructures and shapes. However, the heat evaporation in curved pipe areas was very high due to the absence of cooling fins.

In their study, Khan and Ahmad^15^ utilized CFD-based thermal analyses to predict the heat transfer rate and pressure drop across a radiator. The heat transfer rate and coefficient condition were analyzed to determine the material's lifespan. The material's ultimate strengths were tested in the most complicated heat zone. The use of Nano fluids was found to reduce the transferring conditions. Fins were utilized to identify complicated structures and path vectors in the stream conditions. Additionally, the fins were employed in cooling processes to prevent heat losses in the material.

Jinsiwale and Achwal^16^ conducted a CFD investigation to compare the flow of water and Nano fluid in carburetor pipes. The study used Al_2_O_3_ as the Nano fluid content. The results showed that the Al_2_O_3_ Nano fluid provided better solutions and quicker cooling temperatures than water flow. The cooling fins were evenly spaced in both the water and Nano fluid setups to ensure consistent heat dissipation. The Al_2_O_3_ Nano fluid absorbed the high-gradient temperature from the pipe more effectively.

Senthilraja et al.^17^ conducted a study on the effects of temperature on radiator pipes and cooling techniques using computational fluid dynamics (CFD). While their investigation was primarily based on normal water fluids, they also explored the use of Nano fluids made from aluminum oxide and titanium oxide. The use of water content led to high temperature levels throughout the pipe flow. However, the addition of Nano fluids resulted in decreased temperature levels due to their superior heat conductivity.

The study by Bupesh Raja et al.^18^ investigated thermal conduction between the radiator pipe and fins. The researchers found that the surfaces and shapes of the ends of curved pipes and fin supports played a critical role in achieving good thermal conduction. Heat transfer was primarily achieved through the velocity path from the inlet pipes. The researchers also found that the complicated surface limitations resulted in good shapes for the heated pipe. Moreover, the absence of fin particles resulted in good heat absorption for the curved pipe. The cooling system with fins was found to be complicated, and the researchers formed it based on the adjacent wall temperatures.

Patel^19^ conducted a study on the use of Nano fluids mixed in water to reduce heat energy. The study utilized Al_2_O_3_ and CuO Nano fluids sent across the radiator pipe. Due to the quick convection that occurred inside the pipe, heat energy was transmitted to the fins. The fins absorbed the heat energy, and the temperature was subsequently reduced by the air conditioner. The results of the Nano fluid process were found to be better when compared to the standard fluid process. While fluid water exerted high pressure and velocity, the use of Nano fluids helped to smoothly decrease these values.

From the literature study, the arrangement of fins with the layer-by-layer process was identified. The novelty of this study explores the influence of nano-fluid properties and temperature effects on the radiator conditions. The fluid flow results show a favourable comparison with the results of other studies. This methodology proves to be helpful and shows significant progress compared to other research. The comparison results are thoroughly investigated and presented in the conclusion section. The above literatures to identified the problem conditions and exact solution in different techniques.

## CFD simulation process and results

### 3D views of radiator and mesh

**Figure 1:**
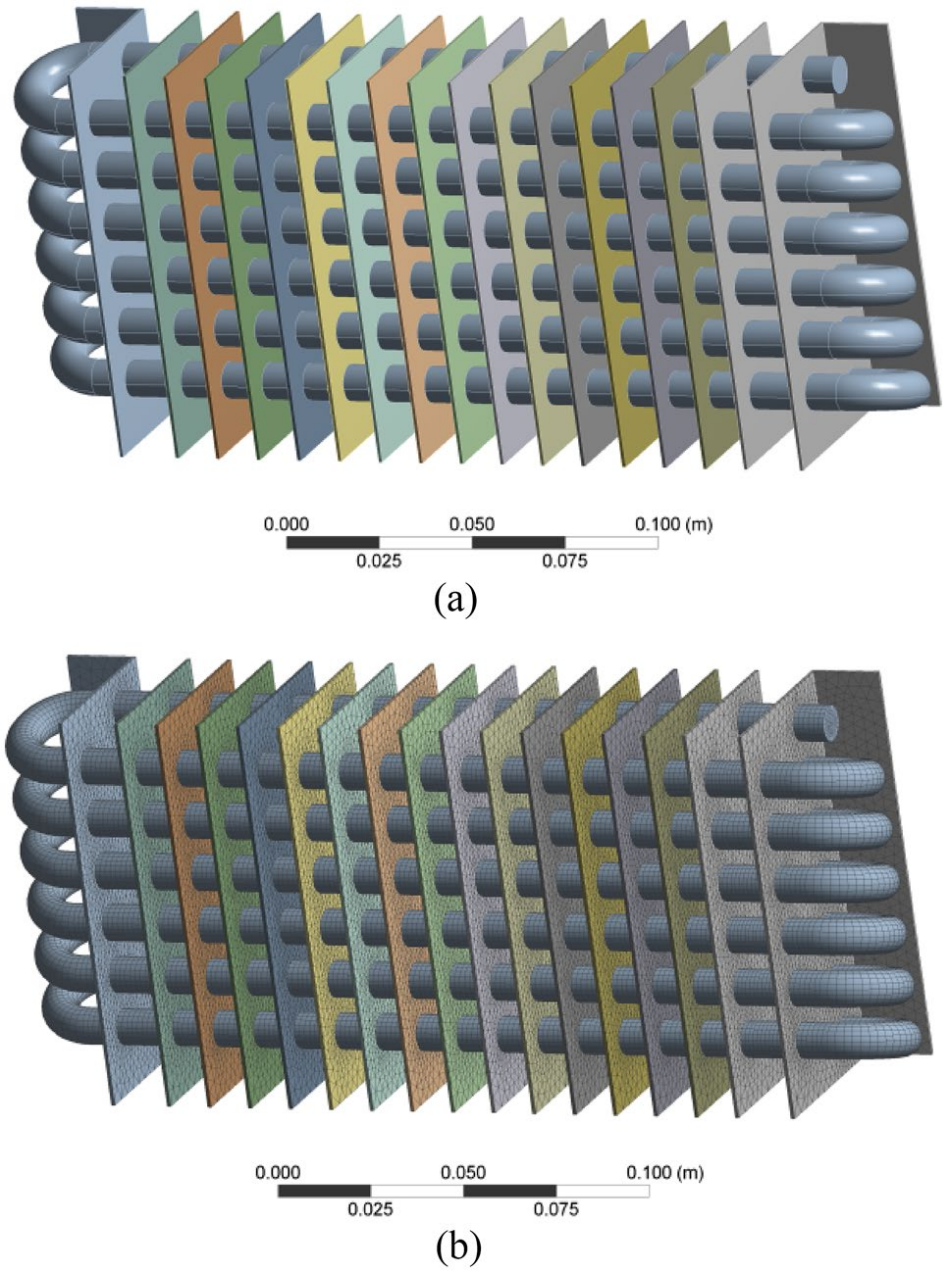
(**a**) 3D model of the radiator. (**b**) 3D model of radiator mesh.

We created a 3D model of a radiator using Creo 4.0, with steel pipelines and composite fins made of a resin composite material. The composite fins were designed to enhance the radiator's cooling performance, and were constructed with equal spacing between fibers. The model is shown in Fig. 1a,b, depicting the radiator and its mesh.

The use of composite fins in radiator cooling systems is a potential solution to address the effects of climate change. Additionally, composites are a cost-effective alternative compared to other standard materials. To create the 3D model of the radiator, Creo 4.0 was used, and tetrahedron meshes were utilized for meshing. The mesh quality was found to improve the material's efficiency, and a triangular shape was formed at the entire radiator surface. Linear effects were observed in curved shapes and conditions, and the diameters of the inlet and outlet valves were the same.

#### Governing equations

The composite parameters appears in the terms of numerical simulation is processed as the thermal behaviours. Governing equation numerical simulation represent the flow variations of the inside thermal boundary conditions and moments. The density of the matrix is represent the constant behaviours in the displacement functions along with the wall temperatures.

1 $$\frac{{d}^{2}T}{{d}^{2}x}=-\frac{hp}{kA}\left(T-{T}_{ambient}\right)$$

$\frac{{d}^{2}T}{{d}^{2}x}$ represent the second derivative of temperature (*T*) with respect to distance (*x*).

*h*—is the convective heat transfer coefficient *b*/*n* the fin and the surrounding fluid (W/(m^2^ K)).

*p*—is the perimeter of the fin’s cross-section (m).

*k*—is the thermal conductivity of the fin material (W/(m K)).

*A*—is the cross-sectional area of the fin (m^2^).

*T*_*ambient*_—is the ambient temperature of the surrounding fluid (K).

2 $$q=\frac{({T}_{1}-{T}_{2})}{{R}_{Total}}$$

*q* = is the heat flux (W/m^2^).

*T*_1_, *T*_2_—are the temperatures at the two ends of the composite laminate (K).

*R*_*Total*_—is the total thermal resistance of the composite laminate (K/W).

3 $$\rho c\frac{\partial t}{\partial T}=\nabla \cdot \left(k\nabla T\right)$$

$\rho$—is the density of the material (kg/m^3^).

*c*—is the specific heat capacity (J/(kg K)).

$\frac{\partial t}{\partial T}$—is the temperature change over time (K/s).

CFD was used to investigate the composite fins. High velocity and low temperature composite elements were being developed. The primary investigation was based on fins. Fins are typically used to lower temperatures and increase velocity.

**Figure 2:**
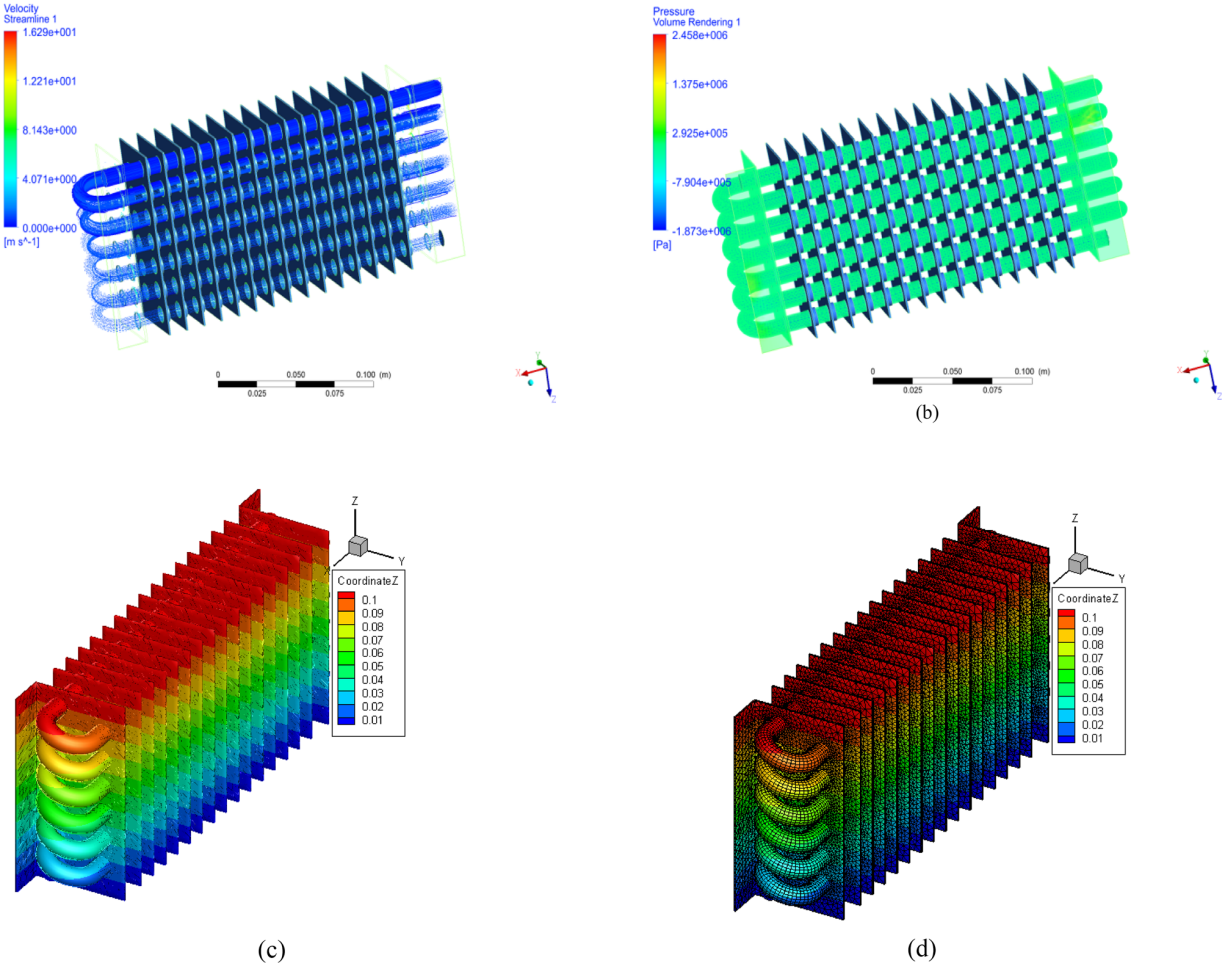
(**a**) Velocity stream line in radiator. (**b**) Pressure volume rending in radiator. (**c**) Heat transfer rate in fins. (**d**) Heat transfer rate with mesh contour.

Temperature effects, velocity variations, and pressure gradients of the radiators studied using CFD are depicted in Fig. 2a,b. However, the most common issue in radiator engines is temperature rise. A composite structure is an excellent way to reduce temperature while increasing velocity.

The inlet valve produced a velocity of 0.04 m/s. The maximum velocity attained was 1.62 m/s. The composite fin reduced the velocity speed at the pipes’ midpoint. The maximum pressure in the inlet valve area was 3.5 MPa. Owing to the pressure, the flow was high in the radiator system. Table 1 represent the dimensions of radiator fins^20^.

**Table 1:** Dimension of radiator.

S. no	Particular	Size
1	Width	0.5–0.6 m
2	Height	0.4–0.7 m
3	Depth	0.025–0.038 m
4	Fin thickness	0.8 mm and 1.0 mm
5	Pipe	1-inch to 3 inches in diameter
6	Angle	130°
7	Number of panels	17

As composite fins are stronger than standard fins, they can withstand high pressure and allow for a continuous flow within the wall. A composite fin attached inside a pipe exerts a pressure of 2.458 Pa. It has a fast transfer rate and facilitates the flow. Figure 2c,d shows that the velocity inside the radiator pipes ranges from 0 to 1.62 m/s, increasing pipe by pipe, with the initial-stage velocity being greater than the end-stage velocity.

### CFD investigation results for nanoparticles

Composite structures have excellent heat flux distribution and surface contact. The mesh and composite fin quality adapt to the linear flow process inside the pipelines. Heat flux distribution is uniform across all pipelines. In curved flow pipes, the common temperature effect is adequate. The good quality of curved areas and surface entities achieve a horizontal temperature effect. Heat is distributed from the inlet valve to the outlet valve. It depicted heat flux variations in vertical directions.

The nonlinear effects and contributions change the temperature effects. Heat flux is reduced directly and the process is transmitted through the composite fins. Composite structures control the most common temperature effect. All the wall surface compositions used to control the heat transfer rate are gradually introduced when the composite fins are introduced. The first fin is introduced at the full rate of heat, and the subsequent fins gradually begin to reduce the temperature rates. The temperature range is controlled using the same method.

### Composite fins with hydrogen gas

**Figure 3:**
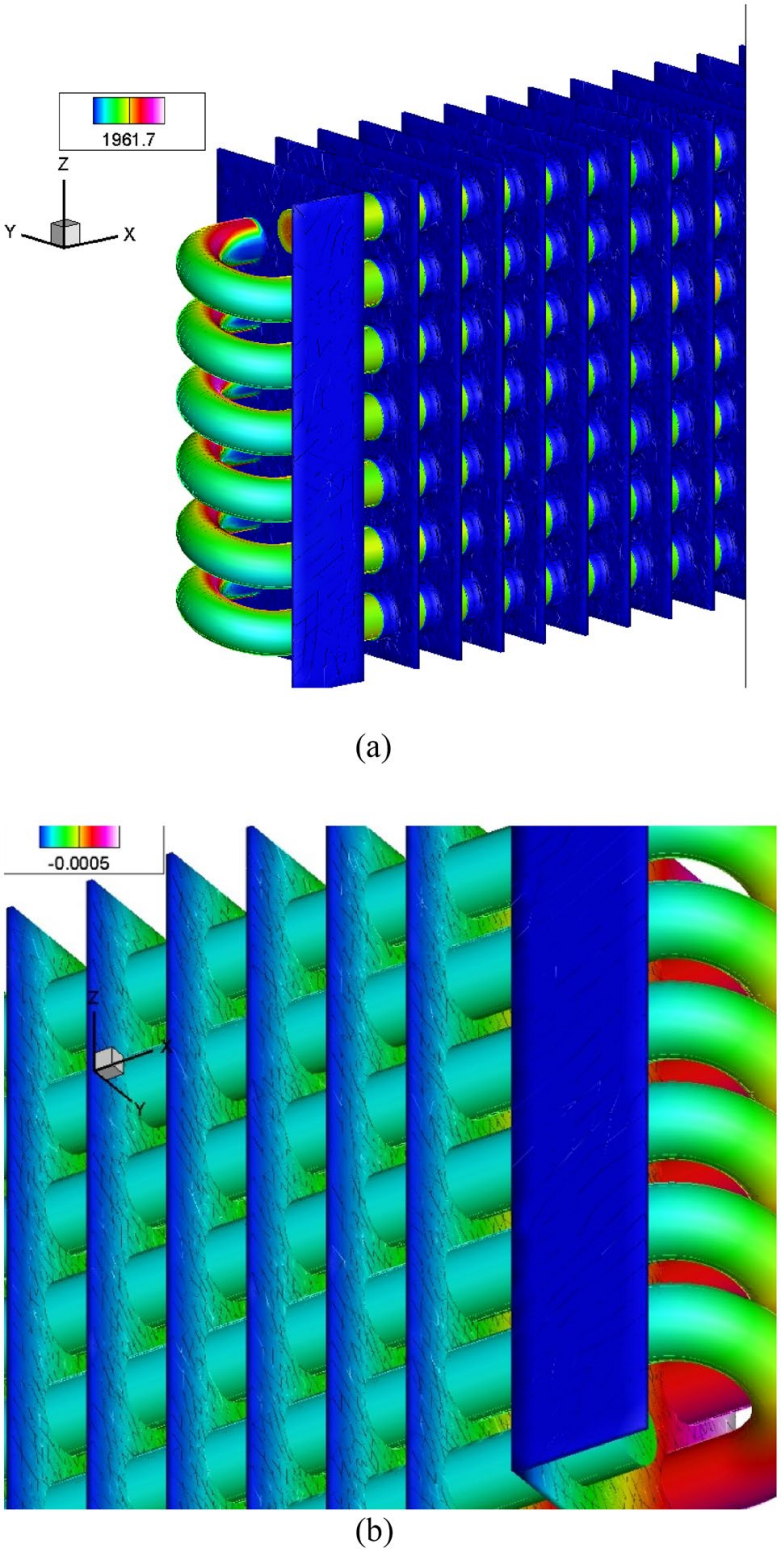
(**a**) Heat transfers and stress distribution. (**b**) Cooling arrangement in fins.

Temperature distribution occurs in the radiator pipelines, and composite fins are used to improve cooling systems. The composite fins absorb radiation effects and heat sources in the streamline pipes. Figure 3a shows the heated areas and stress distribution observed by the composite cooling fins. The initial section shows regions of high-velocity fluid flow. The temperature effects are controlled at the midpoint of the pipeline using composite fins. Heat transfer ranges from 312 to 462 kJ in the pipelines.

Composite fins are effective in controlling the temperature variations in cooling systems due to their short distance. As shown in Fig. 3b, the temperature distribution levels from inlet to exhaust are evenly distributed due to the variations in the pipelines. The nature of the cooling arrangements contributes to achieving good temperature effects. However, the heating effects are most pronounced in the bend areas. When comparing composite fins to steel fins, composite fins show better variations and results.

The thermal distributions are utilized to compute the heat transfer coefficient and cooling process, with the cooling fins proving to be highly effective in reducing temperature variations. The flow process inside the pipes and the heat transfer zones carry stress distributions and effects. The cooling system of the radiator is dependent on the ambient temperature. The curved paths' bending area experiences the highest stress distribution, and the longer pipes operate under nonlinear conditions within the range of distributions.

Temperature ranges and cooling strategies are used to control the ambient air temperature. Composite fins are effective in allowing heated pipe temperatures to escape. The spacing between composite fins is critical in cooling systems. Composite materials transfer heat from the radiator to the fins. When two fins face high-temperature effects, they are neutralized by the composite fin surfaces. The variations in inside and outside gas flow are associated with the coordinate effect.

### Thermal distributions

Two types of inlet fluids are used in radiators: NoX and nanoparticles. The inlet velocity remains the same for both fluids, but there is pressure variance in the radiator system. Nitrogen oxide gas is introduced through the inlet valve to adjust the temperature and atmospheric conditions. When examined using composite fins, the stress level in the appropriate and curved areas was found to be high. The efficiency of NoX gas is high in the inlet boundary conditions. According to studies, the thermal stress range is 2.5% lower than with normal gases.

**Figure 4:**
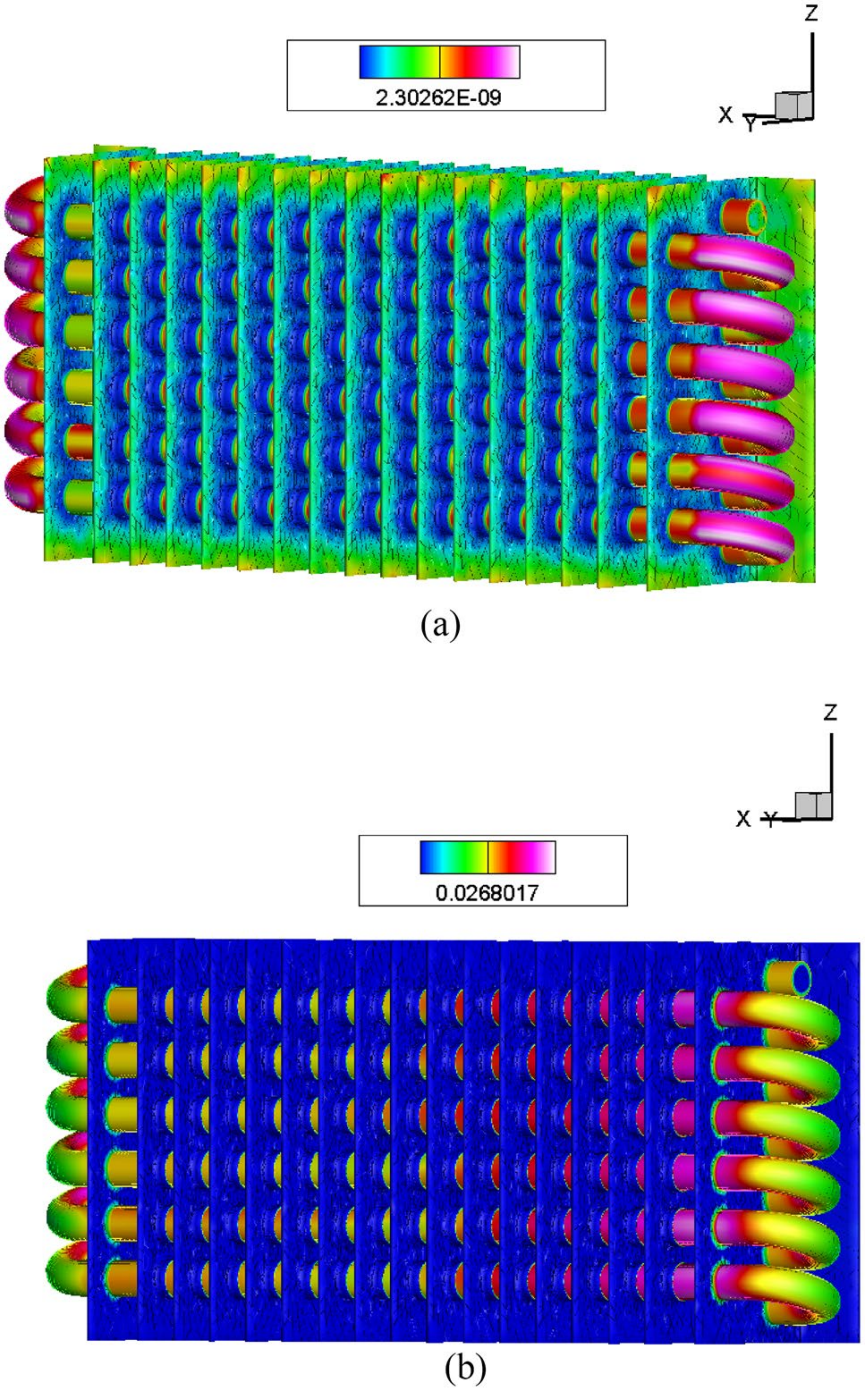
(**a**) Temperature analysis using external air. (**b**) Von Mises arrangements in the radiator.

The stress areas in the curved pipeline joints are the highest compared to other areas. The stress effect at the initial areas is very low compared to the middle of the stress areas. This is because the velocity of the pipeline at the inlet valve is low and gradually increases towards the middle partition. The common effective variations and heat flux are transformed from inside to outside. Figure 4a shows the distribution of stress from inlet to outlet. The composite results of heated conditions and paths are shown in the orientation view. Figure 4b shows how heat is transferred from the pipe to the fins through the composite material.

The cooling system of composite fins is mainly powered by atmospheric air, resulting in low temperature decreases in cooling systems. External radiator fans are used to reduce higher temperature levels caused by air conditioning, which can quickly decrease the temperature effects in composite fins. The vapor operating at high temperatures is characterized by the von Mises stress and liquid processes. The interaction between natural gases and nanoparticles is the most common. Figure 4b shows a von Mises stress value of 0.0268017 MPa. The inlet valve areas have lower von Mises stresses than the exit area. The use of composite fins lowers von Mises stresses and boosts Nano fluid efficiency. Figure 4a provides an overall view of the effect. The nanoparticle process adapts the temperature effects of exhaust gases.

## Results and discussion

The CFD process is used to analyze temperature variations inside the radiator. Temperature variations range from 830 to 910 K. The temperature field is very high at the inlet areas, whereas the curved areas have normal temperatures. The temperature is mostly reduced in the outlet valves. The composite fins achieve the highest temperature reduction in the field ranges. The effectiveness of composite fins is determined by the number of iterations controlled by CFD variations.

### Velocity distributions

The velocity ranges vary from the inlet to the outlet boundary. The effects of velocity distribution are more prominent in the Y-direction. The contour plots arranged in curved beams have an impact on temperature migration. The fluid motion velocity is generated by rotation and ranges from − 1.5 to 1.5 m/s. Common velocity ranges are determined using CFD methods. The velocity path at the curved end is predicted to be higher, whereas the sides of the pipeline exhibit normal effects.

**Figure 5:**
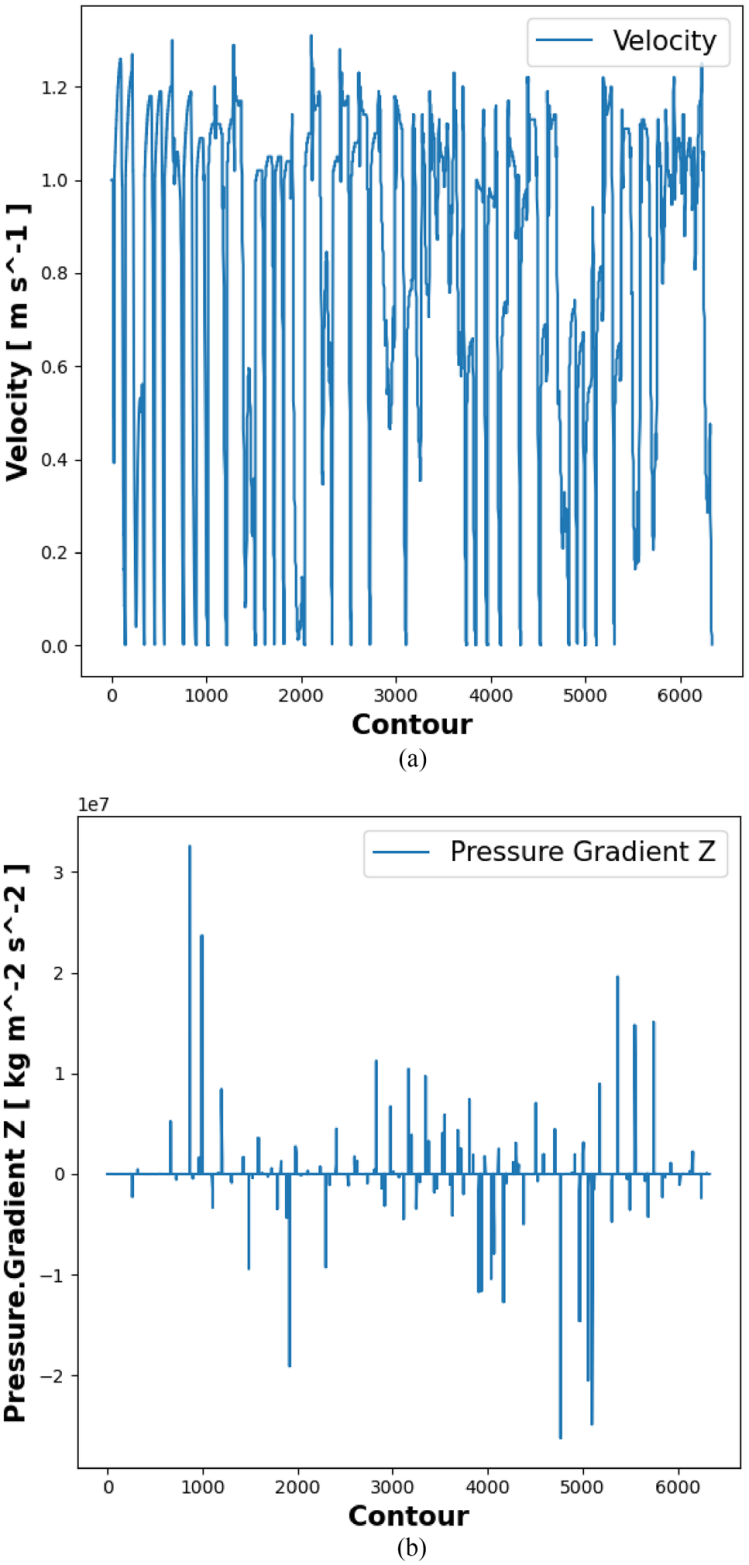
(**a**) Velocity distribution in Nano fluids. (**b**) Pressure gradient in Z-contours.

The required flow rate for the initial iteration is very low, while the middle iteration range is greater than that at the end. Figure 5a displays the streamline of the composite fins. The counter effect is determined by the temperature process. Figure 5b illustrates the impact of temperature distribution. CFD arrangements are used to manage the line path economically. The streamline effect is rearranged in the flow of radiator pipes. Temperature variation affects the streamline in hydrogen gas fluids and Nano fluids.

### Pressure gradient

**Figure 6:**
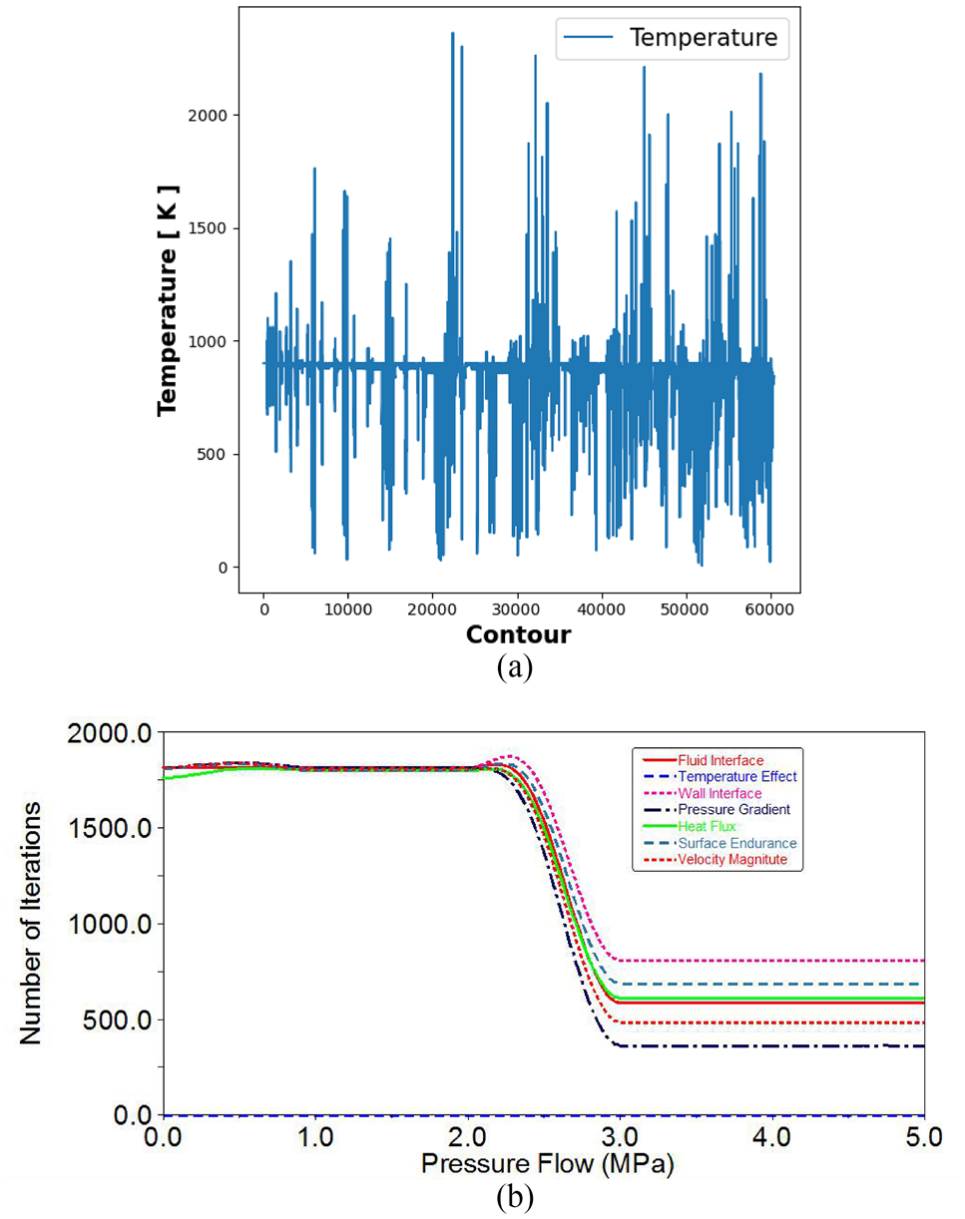
(**a**) Temperature distributions in fins. (**b**)Variations in pressure flow in the pipe with Nano fluid particles.

On the Z-axis were compared to determine the effect of velocity distribution. The contour iterations were used to study the velocity grid under nonlinear conditions, as shown in Fig. 5b. An Ansys CFD simulation was used to produce the velocity distribution path line. The number of iterations predicted the surface paths and bonding efficiency, as shown in Fig. 6a. The velocity magnitude and direction were achieved by a large number of structures and thermal behaviors.

CFD simulations were used to determine the velocity of nanoparticles. The vector path showed the velocity deviation. The velocity ranges between − 1.5 and 1.5 m/s. The peak variation began at the inlet pipeline and progressed to the outlet pipe. The curved lines had a high efficiency. The initial velocity was high and reached the outlet in lower percentages than the middle partition. The flow was adapted to the behavior of stream path lines and surfaces.

### Temperature effect and analysis

The frequency paths of flow vibrations were derived from good contacts and nonlinear surfaces, as shown in Fig. 6a. The pressure gradients appeared in the radiator pipelines and flow processes. The conditions were generated by the curved pipeline design and strategies. Figure 6b shows the greatest pressure gradient, representing a pressure level of 4 × 10^7^ MPa. The pressure gradient is greater in the z-contours. The maximum pressure is formed by the inlet pipeline behavior. The pressure in the curved pipeline is greater at the end stage, which is because composite fins reduce pressure rate and contact.

The pressure stream path for Nano fluids is shown to be a good flow path. The results are displayed in a graph, which shows the variation in pressure from the inlet to the outlet in the radiator simulation. The pressure is initially normal, but it appears high when it reaches the exit. In the curved pipelines of the radiator, the maximum pressure rate is 4.5 × 10^7^ MPa. The pressure rates inside the pipeline processes are increased by the composite fins and ranges.

### Pressure variation analysis

**Figure 7:**
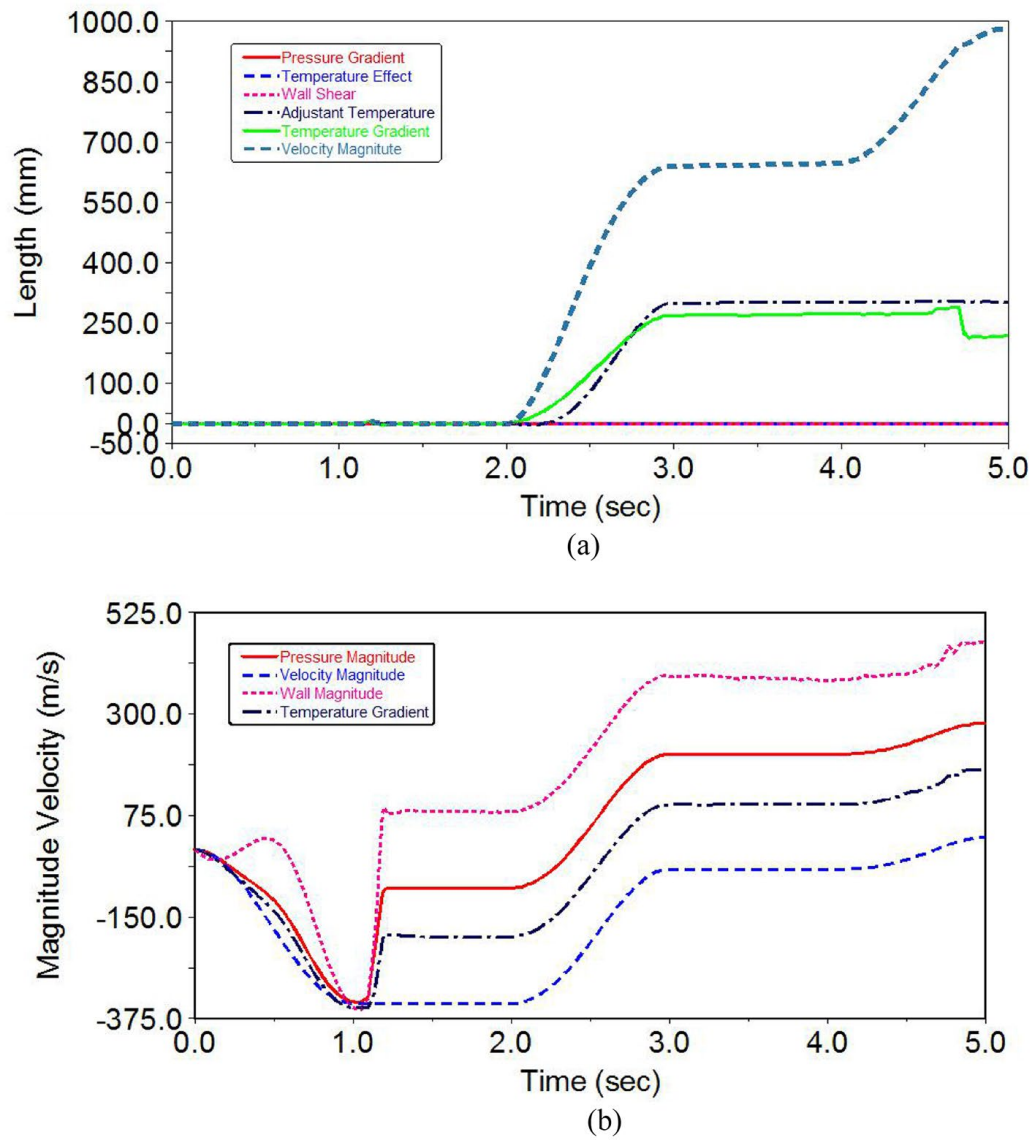
(**a**) Time vs length parameter with pressure distribution. (**b**) Velocity magnitude in a radiator pipe.

A comparison was made between natural gas and nanoparticle-mixed gas flow conditions in composite fins. The maximum pressure flow range was found to be 5 MPa, with the most efficient ranges being up to 2 MPa as determined by CFD analysis. However, pressure variations increased from contact to surfaces when passing through the curved pipe. The linear effects and conditions were adapted to the coordination of the given path. Figure 7a shows all the effects and variations, with CFD values used to analyze the controlled range. The heat flux surfaces caused a quick effect on the flow conditions.

The initial temperature range observed in the radiator is 800–1000 K, while the final temperature is 1850 K, indicating a significant increase. The maximum temperature deviation occurs in the middle curved pipeline stage, which is attributed to the cooling system provided by the composite fins. It is important to note that this cooling system is more expensive compared to standard fins. The thermal distribution effects of natural gas and nanoparticle-mixed gas were determined using CFD results.

CFD was used to investigate the total lengths of pipes and the fluid passing time of nanoparticles across all regions. The simulation system included the structural results and surfaces of the radiator. As the time setup increased, the passing time of fluid particles gradually increased in the radiators, and there was a variation in the passing time depending on the length of the curved pipe. The time parameter also varied according to the region of contact with the composite fins. The temperature distributions greatly varied across the composite fin element. Figure 7b displays a graph of the passing time as a function of length.

Pressure gradient, temperature effect, wall shear, adjutant temperature, temperature gradient, and velocity magnitude are all components of pressure. The initial stage pressure is normal; however, it may increase when it reaches the curved pipe line. The simulation time was also increased at a constant rate. In the entire CFD simulations, the wall effect, pressure gradient, and temperature effect did not change with the increase in time. Compared to previous work, this research achieved 2.5% higher efficiency in cooling systems. This indicates that the composite fin-attached radiators regulate temperature effects and do not cause any loss effect.

**Figure 8:**
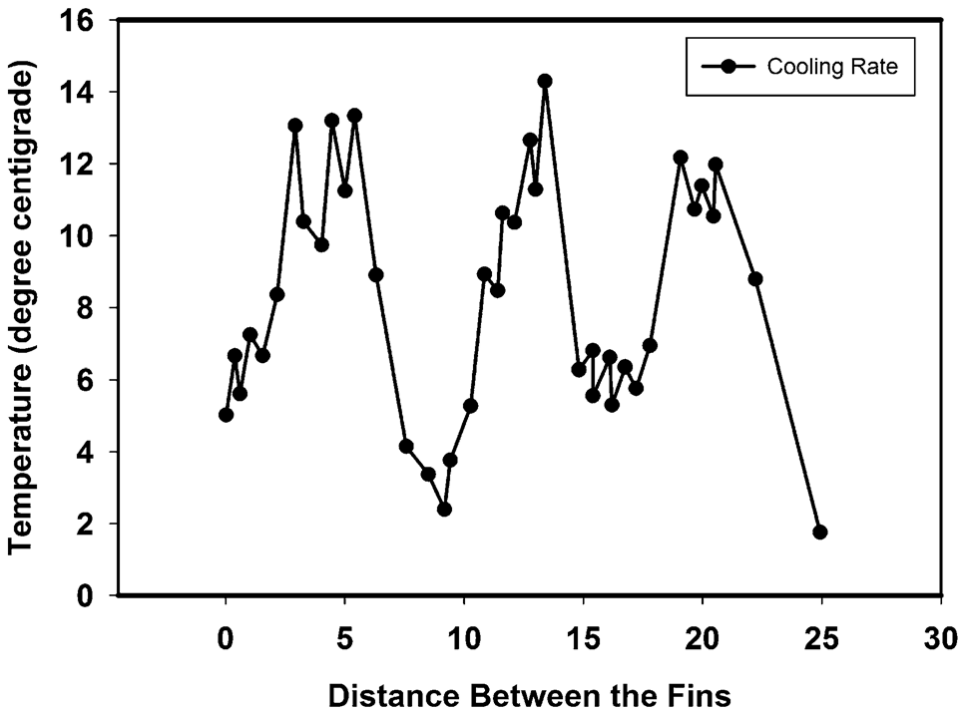
Cooling systems in between the fins.

The composite radiator was designed with a flow process that adapted to good contact interpretations. CFD investigations were used to examine the velocity magnitude results, and the curved pipe elements demonstrated good performance in the nanoparticle process, as well as cooling systems are shown in Fig. 8. The cooling rate of radiator range from 6 to 14 °C. The limitations of surfaces were overcome by the effect of the nonlinear boundary region. Magnitude, velocity, and time were used to determine the limitations at the end of the stage. Time is an important factor in CFD simulations. The effect of heat transfer flow in radiator structures with fins was determined by CFD investigation, and the composite fins showed complete control.

## Grid independent study

Velocity determines the distribution of force from inlet to outlet. The diameters of the inlet and outlet valves were the same, but the curved pipe variations differed. The pressure gradient in the *x*-direction was used to control the linear motion. The CFD investigations examine the linear process and frequency. The concept of linear distribution changes over time. In this study, the force distribution formed evenly throughout the radiator pipeline system. The composite fins cooling control system lowered temperatures in the radiator pipes.

**Figure 9:**
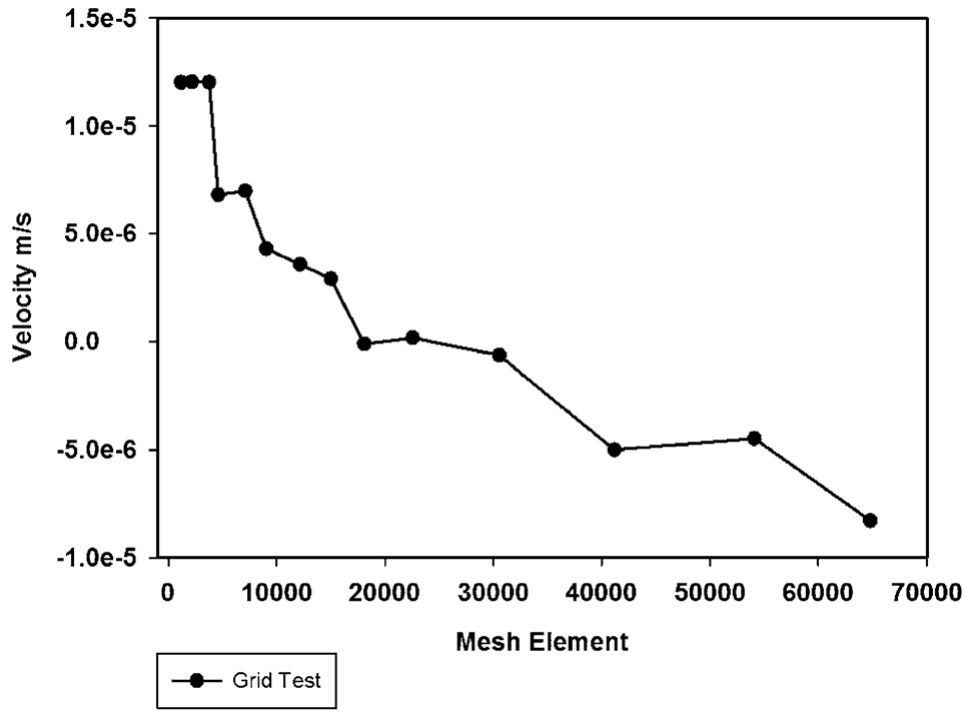
Grid independent test comparison for error predictions.

Figure 9 illustrates the force action process inside the radiator pipeline system for error predictions. The frequency distribution level gradually increases across all pipeline conditions. The force is formed in all directions and is shown by the velocity gradients.

**Figure 10:**
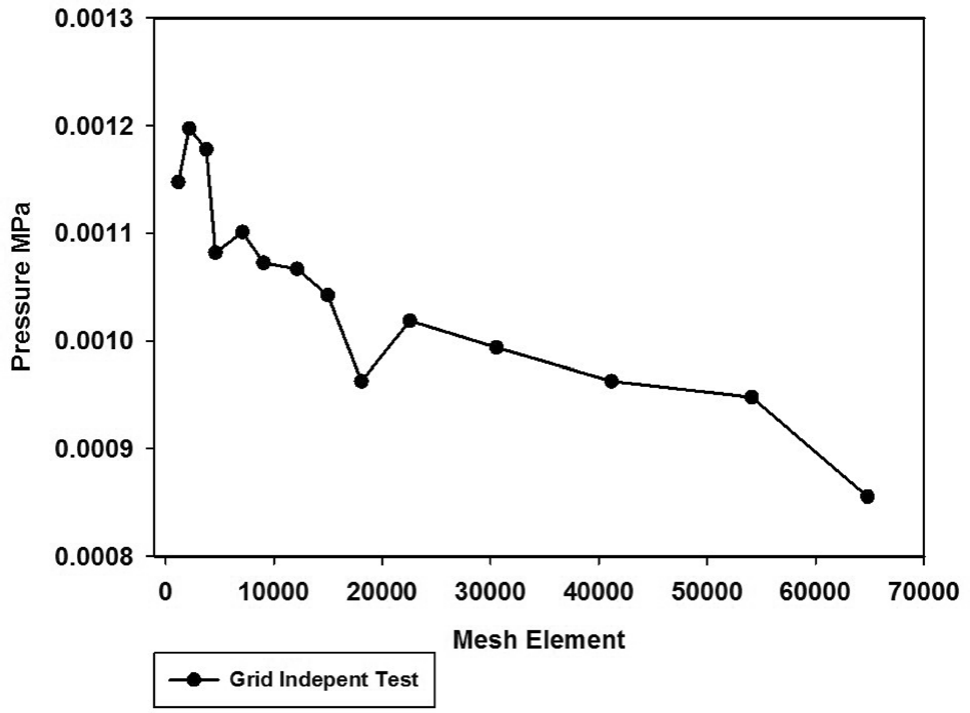
Pressure based error analysis compare with grid independent study.

Figure 10 is shown grid independent study of radiation effects in the radiator setup are controlled by both the gravitational effect and the duration of time. The thermal effects of the composite fins durable system were controlled by adjusting the velocity factors^20–22^.

## Conclusions

Composite fins have proven to be effective cooling solutions in radiator simulations for thermal experiments, as they can efficiently transform heat energy. In this investigation, we controlled heat energy using composite fins. Computational Fluid Dynamics (CFD) simulations were conducted to assess the thermal properties and cooling systems employing a Nano fluid. The velocity at the inlet of the radiator pipeline varied due to curved pipeline paths, leading to fluctuating flow velocities throughout the pipeline processes. Additionally, the heating effects and material properties of the composite fins were considered. The thermal energy passes through the inlet and outlet valves, resulting in force distributions and linear variations.

- The simulation results indicate a von Mises stress of 0.0268, a change in the composite fin heat transfer rate to 2.301, and a heat transfer temperature rate of approximately 900 K. The pressure gradient also reached 2.5 MPa.
- At a pressure flow of 2 MPa, there were signs of stress variations and heat losses. The total pipe flow covers a distance of 1000 mm with a velocity magnitude of 15 m/s from the beginning to the end of the stages.
- In the simulation, the entire pipe flow is subjected to a force of 3500 N to be completed within 5 s.
- Analytical calculations of Rayleigh deviation values improved by 1.97% compared to the experimental results.
- This investigation has shown that variations in flow with Nano fluids are highly dependent on the interaction between the Nusselt number and volume fractions. The presence of composite fins reduces the heat transfer rate within the fluid process, as they are designed to decrease the thermal efficiency of the heat exchanger.
- Thermal properties can be enhanced by adjusting the pressure and changing the time. Stress and strain rates constantly change due to input variations. It is crucial to construct the radiator setup correctly to obtain accurate simulation results.
- The von Mises stress results demonstrate the thermal efficiency of hydrogen gas with Nano fluid properties. Future work will involve improving the design of composite fins with holes and incorporating nanoparticles throughout the simulation flow. Model outcomes will also be compared with experimental findings.

## Future work

The design and structure of the fins will be modified, and a comparative review will be conducted with reference to previous studies. The simulation work will be done with fins that have holes, and the results of this comparison will be analyzed. Additionally, the comparison results will be verified by simulating fins with holes and Nano fluid particles.

## Data Availability

The datasets used and/or analysed during the current study available from the corresponding author on reasonable request.

## Abbreviations

Inlet: The entrance or starting point of the flow
Outlet: The exit or endpoint of the flow
Streamline: A line that represents the instantaneous direction of the flow velocity at each point in a fluid flow field
Stress distribution: The pattern or distribution of internal forces within a material or structure
Meshed view: A representation of the object or structure using a grid-like mesh to discretize the geometry for analysis
MPa: Mega pascal
N: Newton
CFD: Computational fluid dynamics
K: Kelvin
Pa: Pascal
