# Author Correction: Enhancing automotive cooling systems: composite fins and nanoparticles analysis in radiators

**DOI:** 10.1038/s41598-024-56013-5

**Published:** 2024-03-05

**Authors:** R. Ramesh Kumar, K. Karthik, P. V. Elumalai, R. Elumalai, Davannendran Chandran, E. Prakash, Nasim Hassin

**Affiliations:** 1 Vel Tech Rangarajan Dr.Sagunthala R&D Institute of Science and Technology, 400 Feet Outer Ring Road Avadi, Chennai, Tamil Nadu India; 2 Department of Mechanical Engineering, Aditya Engineering College, Surampalem, Andhra Pradesh India; 3 Department of Mechanical Engineering, Vellore Institute of Technology, Chennai, India; 4 Department of Mechanical Engineering, Universiti Teknologi PETRONAS, Seri Iskandar, Malaysia; 5 Department of Mechatronics Engineering, Rajalakshmi Engineering College, Chennai, India; 6 Department of Mechanical Engineering, Mettu University, Metu, Ethiopia

Correction to: *Scientific Reports* 10.1038/s41598-024-52141-0, published online 13 February 2024

The original version of this Article contained an error in Affiliation 5, which was incorrectly given as ‘Department of Mechanical Engineering, MLR Institute of Technology, Hyderabad, India’. The correct affiliation is listed below:

Department of Mechatronics Engineering, Rajalakshmi Engineering College, Chennai, India.

The original Article has been corrected.

